# Case Report: Multiple mesenchymal hamartomas showing spontaneous motility in the nasopharynx

**DOI:** 10.3389/fonc.2025.1480087

**Published:** 2025-02-28

**Authors:** Tetsuya Kinoshita, Masamitsu Kono, Ryuta Iwamoto, Masayoshi Hijiya, Saori Takeda, Makiko Ohtani, Mikako Doyama, Daichi Murakami, Shunji Tamagawa, Shin-Ichi Murata, Muneki Hotomi

**Affiliations:** ^1^ Department of Otorhinolaryngology-Head and Neck Surgery, Wakayama Medical University, Wakayama, Japan; ^2^ Department of Human Pathology, Wakayama Medical University, Wakayama, Japan

**Keywords:** hamartoma, nasopharynx, multiple occurrences, spontaneous motility, interstitial cell of Cajal

## Abstract

Hamartomas are classified as either epithelial, mesenchymal, or epithelium-mesenchymal mixed types. They occur extremely rarely in the nasopharynx. A 58-year-old man had multiple tumors with repeating contraction and relaxation in the nasopharynx shown on endoscopy. One of the tumors was endoscopically resected under general anesthesia for histological examination and was finally diagnosed as mesenchymal hamartoma. Smooth muscle tissue is sometimes confirmed in mesenchymal hamartomas, but spontaneous contraction in nasopharyngeal hamartomas is rare. In this case, coexistence of muscle tissue, Cajal interstitial cell-like cells, and ganglion cells was thought to be a mechanism of spontaneous motility of the tumors. Aberrant or residual foregut tissue in the nasopharynx due to embryological factors may be a contributing factor to the multiple occurrences.

## 1 Introduction

Various types of tumors, both benign and malignant, can occur in the nasopharynx. In planning appropriate, precise strategies for diagnosis and treatment, histological diagnosis can provide valuable information in addition to endoscopy and imaging (1). Accumulating evidence of the characteristics related to the diagnosis and treatment of nasopharyngeal benign tumors is therefore thought to be necessary, especially in the case of rare individual diseases.

Hamartomas, which are typically characterized as a single smooth-surface mass, are rare in the nasopharynx. Here, we present a case of multiple hamartomas in the nasopharynx with spontaneous contraction and relaxation.

## 2 Case description

A 58-year-old man became aware of an abnormal feeling within the pharynx 2 years before the current presentation but did not seek medical consultation. He was eventually referred to our hospital for diagnosis and treatment when tumors in the nasopharynx were incidentally discovered during laryngoscopy in a nearby hospital for 1-month hoarseness caused by a vocal cord polyp.

**Figure 1:**
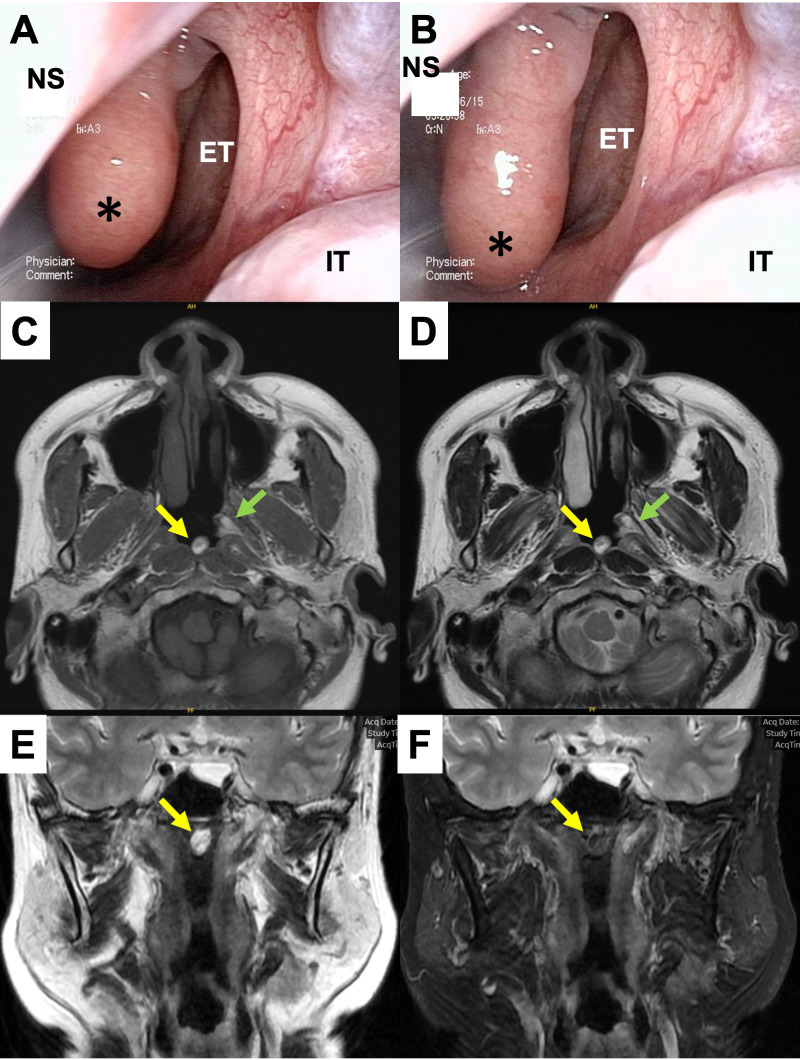
Preoperative findings. **(A, B)** Endoscopic findings in the left nasopharynx. A smooth-surfaced tumor originating from the nasopharyngeal superior wall was spontaneously contracting **(A)** and relaxing **(B)**. *, the tumor; NS, nasal septum; ET, Eustachian tube; IT, inferior turbinate. **(C–F)** MRI findings at the level of the nasopharynx. The tumor showed high intensity including partial iso intensity in both T1 **(C)** and T2-weighed images **(D, E)**. In a fat-suppressed image, the intensity of the tumor turned low signal **(F)**.

Endoscopic examination at our hospital confirmed two tumors moving in the nasopharynx other than vocal cord polyp. They were located upon the superior wall of the nasopharynx (**Figures 1A, B**) and the pharyngeal orifice of the Eustachian tube, respectively. They both had smooth surfaces and spontaneously contracted and relaxed without specific stimulation (**Supplementary Video 1**). Lesions with high intensity and areas of partial low intensity were shown in both T1- and T2-weighted plain magnetic resonance imaging (MRI) (**Figures 1C–E**). T2-weighted images with fat suppression showed low-intensity signals that were consistent with the tumor sites (**Figure 1F**).

**Figure 2:**
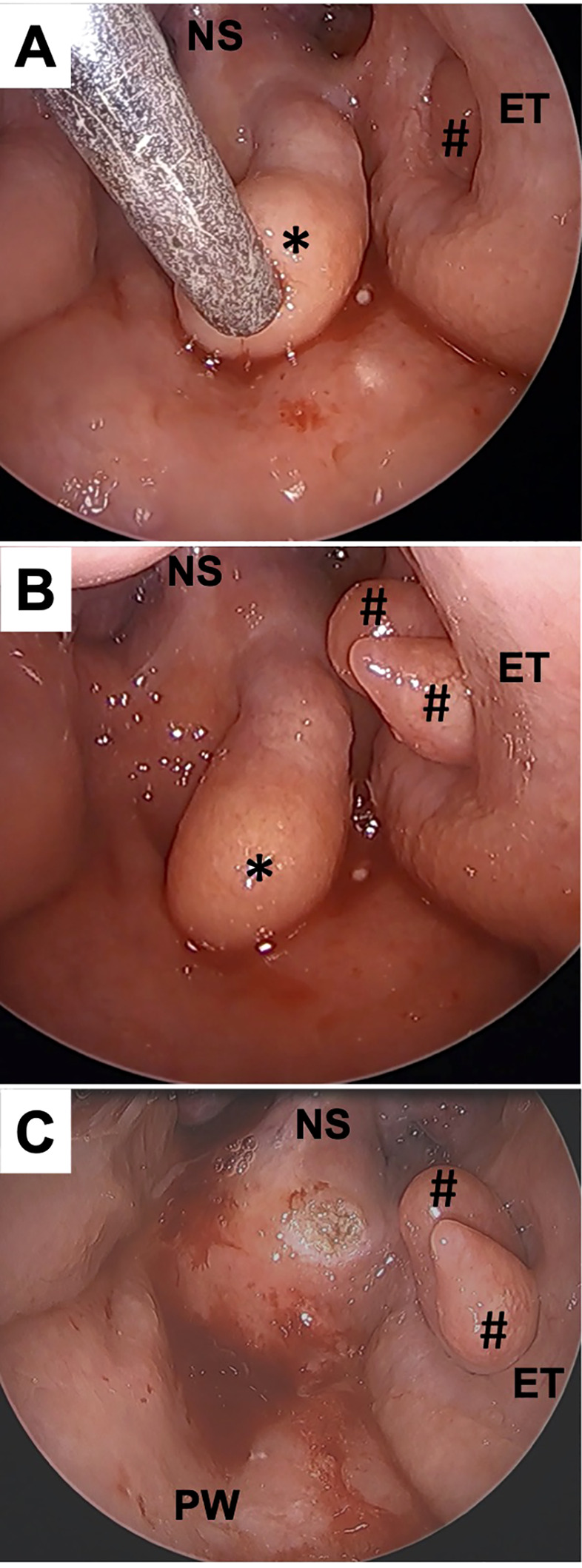
Intraoperative findings. A 70° rigid endoscope was trans-orally inserted to the oropharynx. Multiple tumors were observed in the nasopharynx. The tumor in the Eustachian tube showed spontaneous contraction **(A)** and relaxation **(B)**. The tumor arising from the superior wall (*) was resected with a coblator without bleeding **(C)**. #, the tumors in Eustachian tube; NS, nasal septum; ET, Eustachian tube; PW, posterior wall of the nasopharynx.

Intraoperative biopsy was performed under general anesthesia with a muscle relaxant for a definitive diagnosis. The base of one of the tumors was shown on the left side of the superior wall of the nasopharynx and was moving back and forth. The other, located in the left pharyngeal orifice of the Eustachian tube, was moving in and out of the Eustachian tube (**Figures 2A, B**) (**Supplementary Video 2**). The tumor located on the superior wall was resected at the base by a coblator set at power level 7 for ablation mode (Smith & Nephew Japan, Tokyo, Japan) (**Figure 2C**). The tumor located in the Eustachian tube appeared to be either a single lobulated mass or multiple masses. Importantly, to avoid permanent disorder of Eustachian tube function, we decided in this case to preserve the tumor in the Eustachian tube.

**Figure 3:**
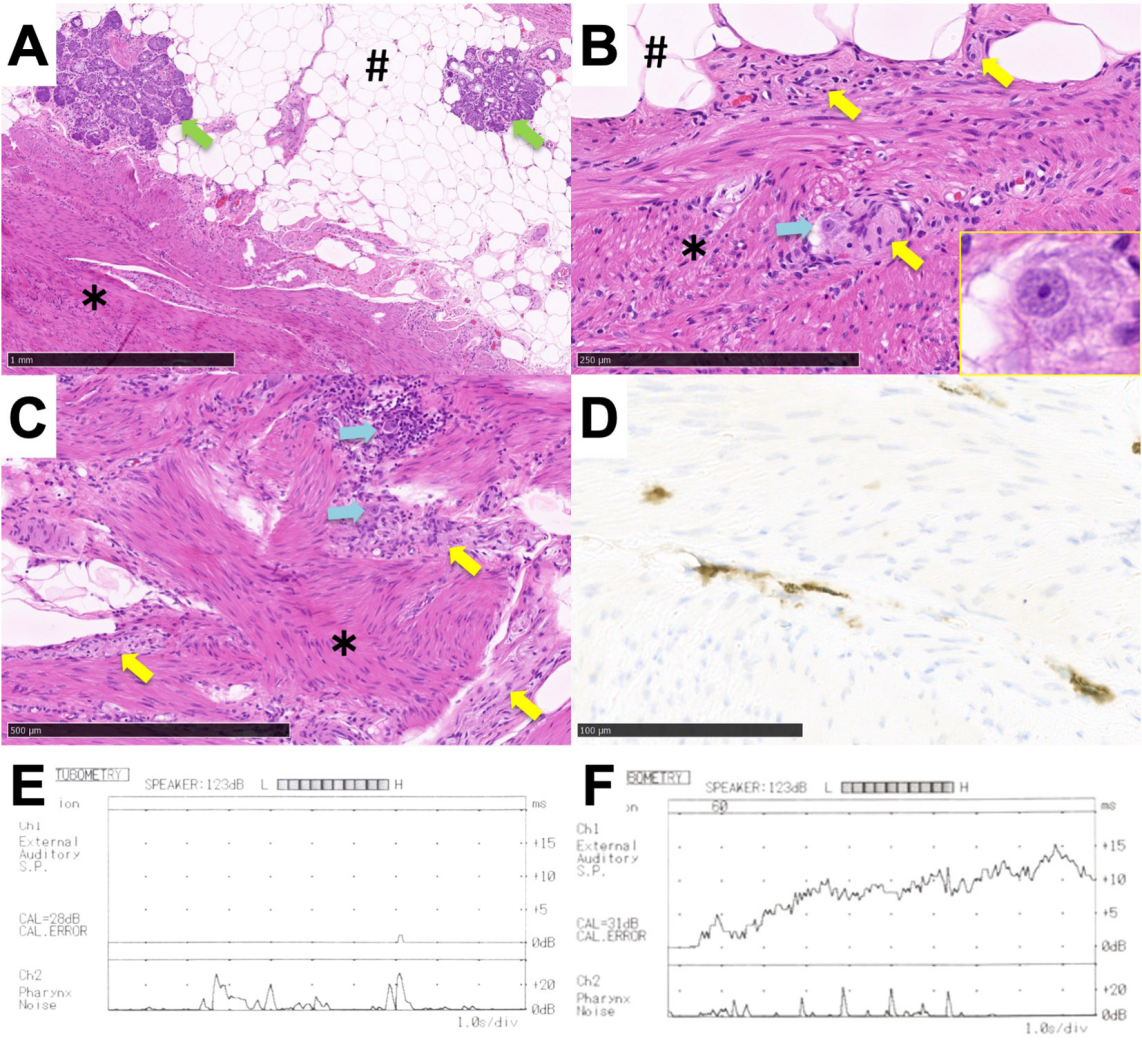
Postoperative findings. **(A–D)** Histological findings of the tumor. **(A–C)** Image of H&E staining. **(A)** Lower magnification (×40). **(B)** Higher magnification including nerve fibers with ganglion cells (×200). Inset figure showing the ganglion cell. **(C)** Higher magnification of muscle tissue showing a myofibrillar disarray (×100). **(D)** Image of immunostaining with anti-KIT antibody with higher magnification (×400). CD117-positive cells were detected in the muscle tissue. #, fat tissue; *, muscle tissue, green arrow; nasal glands, yellow arrow; nerve fibers, blue arrow; ganglion cells. **(E, F)** Left Eustachian tube function test on different test days. Sonotubometry was performed on different test days. **(E)** Finding with Eustachian tube stenosis. **(F)** Finding with patulous Eustachian tube.

Pathological findings included various tissues mixed inside the tumor, such as adipocytes, nasal glands, smooth muscle fibers, and parts of the nervous system, including ganglion cells (**Figures 3A, B**). The smooth muscle tissue presented myofibrillar disarray (**Figure 3C**). CD117-positive cells were shown inside the muscle tissue in the tumor by immunostaining with anti-c-KIT antibodies (**Figure 3D**). According to these findings, our final diagnosis was mesenchymal hamartoma.

Two years have passed without recurrence of the remnant tumor in the nasopharyngeal superior wall. Although the size of the tumor in the Eustachian tube remained unchanged, the patient developed otitis media with effusion. Sonotubometry (JK-05A, RION Co., Ltd. Kokubunji, Japan) confirmed both patulous Eustachian tube and stenosis of the Eustachian tube, depending on the test date (**Figures 3E, F**). The patient ultimately underwent insertion of a ventilation tube in the left ear for recurrent otitis media with effusion. He provided informed consent to the publication of the details of his case.

## 3 Discussion

We presented a rare case of hamartomas in the nasopharynx with pathological evaluation and literature review. A hamartoma is a type of tissue congenital abnormality in which a part grows abnormally due to a congenital abnormal mixture of tissue components (1). They most commonly occur in the lungs, kidneys and intestines, and rarely in the upper respiratory tract.

As the differential diagnosis of hamartoma, we considered benign disease, malignant disease in the nasopharynx, and physiologic hypertrophy of the adenoid. Intracranial lesions such as pituitary tumors were denied because the skull base was intact. MRI is thought to be helpful for qualitative diagnosis of nasopharyngeal tumors. Typical findings of various nasopharyngeal tumors are summarized in **Table 1**. Our patient’s tumors contained a large fatty component, so it was difficult to differentiate them from lipoma by plain MRI. Lipoma was ultimately denied owing to their motility. Other benign diseases that are known to show masses in the nasopharynx, such as juvenile angiofibroma, Tornwaldt cyst, papilloma, minor salivary gland tumor, and glomus tumor, were denied by their appearance, image findings, and motility. Despite the differences in appearance, malignant diseases such as nasopharyngeal cancer and malignant lymphoma should still be excluded by careful biopsy.

**Table 1:** Typical MRI findings in the nasopharyngeal tumors.

Tumor	T1-Weighted Imaging	T2-Weighted Imaging	Contrast Enhancement	Other features
Nasopharyngeal carcinoma	Iso- to hypointense	Hyperintense	Strong, heterogenous	Restricted diffusion
Adenoid vegetation	Iso- to slightly hypointense	Hyperintense	Mild to moderate, sometimes heterogenous	
Lipoma	Homogeneously hyperintense	Homogeneously hyperintense	No enhancement	Complete suppression in fat suppression
Juvenile angiofibroma	Iso- to hypointense with hyperintense area (hemorrhage)	Hyperintense with flow voids	Intense, homogeneous, with flow voids	No restricted diffusion
Tornwaldt cyst	Iso- to hyperintense (fluid or proteinaceous content)	Hyperintense (fluid)	None (cyst contents)	
Papilloma	Iso- to hypointense	Hyperintense with possible convoluted cerebriform pattern	Moderate to intense, heterogeneous	
Minor salivary duct tumor	Iso- to slightly hypointense	Homogeneously hyperintense	Homogeneous or lobulated enhancement	None of diffusion restriction
Glomus tumor	Iso- to hypointense with hyperintense area (hemorrhage)	Hyperintense with salt-and-pepper appearance (flow voids)	Intense, heterogeneous	
Hamartoma	Iso- to hypointense with potential hyperintense area (fat/hemorrhage)	Hyperintense, often heterogeneous	Variable (homogeneous or heterogeneous)	Fat suppression sequences may highlight adipose components

We reviewed the literature on hamartoma arising in the nasopharynx (**Table 2**). The mean age of the 16 patients in the literature (including the current patient) was 32.2 years old, ranging between 0 and 77 years old. The chief complaints were related to obstructive symptoms due to the tumor growth, such as nasal obstruction, snoring, and dyspnea (43.8%), which is thought to reflect the location of the tumors obstructing the upper airway. Three patients (18.8%) were asymptomatic, and their hamartomas were found incidentally. Common sites of occurrence were the lateral wall (37.5%) and the superior wall (31.2%) of the nasopharynx (2–16).

**Table 2:** Summary of previous reports written in English of nasopharyngeal hamartoma.

Age	Sex	Symptom	Site of occurrence	Reference
0	M	dyspnea	superior wall	(2)
3	F	rhinorrhea	posterior wall	(3)
15	M	nasal obstruction	lateral wall	(4)
19	F	cough	lateral wall	(5)
19	M	nasal obstruction, rhinorrhea	superior wall	(6)
22	F	snoring	posterior wall	(7)
24	M	asymptomatic	soft palate	(8)
26	M	nasal obstruction	soft palate	(9)
27	F	headache	lateral wall	(10)
32	M	nasal obstruction	superior wall	(11)
42	F	asymptomatic	lateral wall	(12)
47	M	headache	superior wall	(13)
48	M	nasal obstruction, rhinorrhea	posterolateral wall	(14)
56	M	aural fullness	eustachian tube	(15)
58	M	asymptomatic	superior wall, eustachian tube	current case
77	F	cheek swelling	lateral wall	(16)

There are two endoscopic approaches to the tumor: trans-nasally and trans-orally, and the goal of this surgery is thought to be achievable by both of them. An advantage of the trans-nasal approach is that it is performed with a 0° endoscope, which allows for linear manipulation using nasal endoscopic equipment. Conversely, an advantage of the transoral approach is that the entire nasopharynx can be observed without being affected by the nasal septum. In this case, nonlinear operation under a 70° oblique scope was required, so we introduced a coblation system that allowed ablation, hemostasis, and suction with a single bendable wand.

Nasopharyngeal mesenchymal hamartoma with spontaneous contraction has been previously reported (10). The authors speculated that the tumors contract and relax due to the systematic arrangement of muscle fibers. In the current case, however, the muscle fibers in the tumor were in disarray and could not be explained by that mechanism. We evaluated the presence of CD117-positive interstitial cell of Cajal (ICC)-like cells, along with the muscle tissue, as a potential contributing factor to the contraction and relaxation. ICCs are special interstitial cells that are present in the smooth muscles of the gastrointestinal tract; they are known to have a gastrointestinal pacemaker-like function (17). We also found ganglion cells, which allow for orderly control of peristalsis in the gastrointestinal tract. The spontaneous contraction/relaxation of mesenchymal hamartoma has been suggested to occur due to the presence of muscle tissue, CD117-positive cells, and ganglion cells within the tumor.

We suggest embryological abnormalities as the reason for cells that should be present in the smooth muscles of the gastrointestinal tract being present in the nasopharyngeal tumor. In animal research using mice, the origin of ICCs has been reported to be mesenchymal cells in the intestinal tract (18). Furthermore, the primitive pharynx, which is the developmental origin of the pharynx, exists in human embryonic stages as part of the foregut. These reports give possible explanations of why tumors occurred in multiple areas in the nasopharynx in our patient. With due consideration, we think that the mesenchymal cells of the foregut either migrated into the pharynx during the embryonic period or they were left behind, resulting in the coexistence of ICC-like cells within the tumor along with the muscle tissue. We suggest that this caused the spontaneous contraction and relaxation of the tumor in the current case.

We also evaluated Eustachian tube function in this case. During the interview, the patient had previously mentioned hearing loss on the affected side, suggesting that he had repeated otitis media with effusion due to Eustachian tube dysfunction. Postoperative Eustachian tube function tests revealed stenosis and patulous Eustachian tube, perhaps due to the repeating occlusion and opening of the Eustachian tube by the spontaneous motility of the tumors and damage to the mucous membranes and muscles near the pharyngeal orifice of the Eustachian tube. Although surgical indications for tumors around the Eustachian tube are unclear, a watchful waiting policy may avoid permanent Eustachian tube dysfunction due to adhesions and scarring if malignancy can be definitively excluded.

In conclusion, our patient had nasopharyngeal mesenchymal hamartoma. MRI is suggested to be helpful in differential diagnosis, but it sometimes shows findings similar to those of other benign diseases. Spontaneous motility, as shown in this case, is a rare but specific finding in hamartomas. Immunohistochemical analysis revealed that the spontaneous motility of tumors can potentially be attributed to the coexistence of ICC-like cells and muscle tissue.

## 4 Perspective of the patient

“I was very surprised to see that there were multiple spontaneously moving tumors in my throat. Having the diagnosis gave me peace of mind. I had difficulty in hearing in one side of the ear for a while, but since having a ventilation tube inserted, I can hear as well as before. I will continue to watch and wait for the remaining tumor.”

## Data Availability

The original contributions presented in the study are included in the article/**Supplementary Material**. Further inquiries can be directed to the corresponding author.
